# Effect of Calmodulin-like Gene (*CML*) Overexpression on Stilbene Biosynthesis in Cell Cultures of *Vitis amurensis* Rupr.

**DOI:** 10.3390/plants11020171

**Published:** 2022-01-10

**Authors:** Olga A. Aleynova, Andrey R. Suprun, Alexey A. Ananev, Nikolay N. Nityagovsky, Zlata V. Ogneva, Alexandra S. Dubrovina, Konstantin V. Kiselev

**Affiliations:** 1 Laboratory of Biotechnology, Federal Scientific Center of the East Asia Terrestrial Biodiversity, FEB RAS, 690022 Vladivostok, Russia; aleynova@biosoil.ru (O.A.A.); suprun.hi@gmail.com (A.R.S.); lexxdance@mail.ru (A.A.A.); niknit1996@gmail.com (N.N.N.); zlata.v.ogneva@gmail.com (Z.V.O.); dubrovina@biosoil.ru (A.S.D.); 2 Department of Biochemistry and Biotechnology, Institute of the World Ocean, Far Eastern Federal University, 690090 Vladivostok, Russia

**Keywords:** CML, calcium, stilbenes, transgenic cells, plant cell cultures

## Abstract

Stilbenes are plant phenolics known to rapidly accumulate in grapevine and other plants in response to injury or pathogen attack and to exhibit a great variety of healing beneficial effects. It has previously been shown that several calmodulin-like protein (*CML*) genes were highly up-regulated in cell cultures of wild-growing grapevine *Vitis amurensis* Rupr. in response to stilbene-modulating conditions, such as stress hormones, UV-C, and stilbene precursors. Both CML functions and stilbene biosynthesis regulation are still poorly understood. In this study, we investigated the effect of overexpression of five *VaCML* genes on stilbene and biomass accumulation in the transformed cell cultures of *V. amurensis*. We obtained 16 transgenic cell lines transformed with the *VaCML52*, *VaCML65*, *VaCML86*, *VaCML93*, and *VaCML95* genes (3–4 independent lines per gene) under the control of the double CaMV 35S promoter. HPLC-MS analysis showed that overexpression of the *VaCML65* led to a considerable and consistent increase in the content of stilbenes of 3.8–23.7 times in all transformed lines in comparison with the control calli, while biomass accumulation was not affected. Transformation of the *V. amurensis* cells with other analyzed *VaCML* genes did not lead to a consistent and considerable effect on stilbene biosynthesis in the cell lines. The results indicate that the *VaCML65* gene is implicated in the signaling pathway regulating stilbene biosynthesis as a strong positive regulator and can be useful in viticulture and winemaking for obtaining grape cultivars with a high content of stilbenes and stress resistance.

## 1. Introduction

Stilbenes are part of a vast group of plant polyphenolic compounds with antimicrobial activities synthesized via the phenylpropanoid pathway by a broad range of unrelated plant families [1,2,3]. Plant stilbenes are well-known to confer a variety of health benefits and possess promising pharmacological applications [4,5,6]. Stilbenes are also classified as phytoalexins that rapidly accumulate in plants in response to injury or microbial pathogens and contribute to plant environmental stress resistance [3,7]. *Trans*-resveratrol (*t*-resveratrol) is the most prominent stilbene with a variety of valuable biologically active properties and was widely used in the food and medicine industries [8]. Stilbenes are known to be involved in plant constitutive and inducible defense reactions against plant fungal and bacterial pathogens, nematodes, and herbivores [7,9]. Biosynthesis of natural stilbenoids is activated in response to a number of other environmental stresses, such as ultraviolet (UV) irradiation, wounding, drought, or unfavorable temperatures [3,9].

Although there is a lack of information about the signaling pathways and biochemical events regulating stilbene biosynthesis in plant tissues, it is known that (1) hormone signaling (salicylic acid (SA), methyl jasmonate (MeJA), ethylene, jasmonic acid, and abscisic acid) [10,11,12,13,14,15]; (2) production of reactive oxygens species (ROS) [10,12,15]; (3) stress-induced increase in calcium cytoplasmic concentration [10,11,16,17], and (4) activation of calcium-dependent protein kinases or CDPKs [18,19,20] are implicated in the activation of stilbene biosynthesis in plant tissues. These signaling events eventually lead to the activation of specific transcription factors responsible for the induction of stilbene synthases (STSs) and other stilbene biosynthetic genes [13,21,22]. Furthermore, our recent results demonstrated high responsiveness of plant calmodulin-like protein (CML) genes to the agents stimulating the biosynthesis of stilbenes, including stilbene precursors, SA and MeJa, and UV-C exposure [23], which suggested plant *CML* genes as promising candidates for the activation of plant defense reactions and stilbene production. Thus, it is known that calcium signaling is implicated in the regulation of stilbene biosynthesis in grapevine [3].

Plants encode several classes of calcium sensor proteins, which perceive and decode the alterations in calcium cytoplasmic concentration induced in response to stress and developmental cues [24,25]. The plant calcium sensor proteins include calmodulins (CaMs), calmodulin-like proteins (CMLs), calcium-dependent protein kinases (CDPKs), calcineurin B-like proteins (CBLs), and CBL-interacting protein kinases (CIPKs) [26,27]. Most of the calcium sensor protein subfamilies are encoded by multigene families where the most numerous are *CMLs*, including 50 genes in *Arabidopsis* [28] and at least 54 *CML* genes in grapevine [29]. While the high number and diversity of plant CMLs suggest their importance in plant survival and productivity, the biological functions of this class of plant calcium-binding proteins remain poorly studied.

CML proteins were classified as “sensor relays” lacking “sensor responder” domains and catalytic activity [26,30]. After calcium binding, CMLs undergo conformational changes and interact with their target proteins whose activity is then altered. CMLs transmit the information from the environmental and developmental cues to various downstream biochemical events leading to a proper physiological response. The available research findings show that plant CMLs are involved in the regulation of various developmental processes, such as trichome branching or seed development, and plant abiotic and biotic stress responses [31,32,33,34]. It was shown that the expression of particular *CML* genes was activated in response to pathogen attack [35] or to stress hormones, such as methyl jasmonate or MeJA (AtCML39) and salicylic acid or SA (AtCML43) [36,37,38]. A number of studies show that overexpression of *CML* genes in plants improved plant abiotic stress resistance or contributed to plant–pathogen protection [39,40,41,42,43]. For example, CaCML13 acts positively in pepper immunity against *Ralstonia solanacearum* infection forming a feedback loop with CabZIP63 transcriptional factor [35].

At present, the relationship of plant CMLs and other calcium sensor proteins with plant defense reactions and production of plant secondary metabolites remains almost unexplored. Recently, it was shown that a number of *CML* genes, such as *VaCML52*, *65*, *93*, and *95*, were highly up-regulated in the leaves and cell cultures of wild grape *Vitis amurensis* Rupr. in response to stilbene precursors, stress hormones (MeJA, SA) and UV-irradiation [23], which are known as potent inductors of stilbene biosynthesis. The Amur grape, *Vitis amurensis* Rupr., displays a high level of stress resistance and stilbene content compared to other Vitaceae species and is now commercially cultivated [3,44,45,46]. Vines of *V. amurensis* are used as a resistant rootstock to breed materials for novel cultivars. Elucidating the molecular mechanisms and pathways underlying the stilbene biosynthesis in *V. amurensis* is of high interest.

In the present study, we aimed to investigate and verify the function of the *VaCML52*, *65*, *93*, and *95* genes as potential positive regulators of stilbene biosynthesis in the grapevine by overexpressing the *CML* genes in cell cultures of *V. amurensis*. We also transformed *V. amurensis* with the *VaCML86* gene, whose expression was not essentially changed in response to the stilbene-modulating conditions, with the purpose to use it as an additional control. Results of the present investigation demonstrated that the *VaCML65* gene is a strong positive regulator of stilbene biosynthesis, while the *VaCML52*, *86*, *93*, and *95* genes are not actively involved in this process. The *VaCML65* gene can be useful in viticulture and winemaking for obtaining grape cultivars with a high content of stilbenes and resistance to environmental stresses.

## 2. Results and Discussion

### 2.1. Genetic Transformation and Selection of the VaCML-Transgenic Cell Lines

To establish *Vitis amurensis* Rupr. cell cultures overexpressing the full-length *VaCML52*, *VaCML65*, *VaCML86*, *VaCML93*, and *VaCML95* genes and the control cell line, the V7 suspension culture of *V. amurensis* was incubated with *A. tumefaciens* strains to bear the pZP-RCS2-*VaCML*-*npt*II construct for *VaCML*-transgenic cells or the pZP-RCS2-*npt*II for the control KA0 cell line. Then, we selected transgenic callus cell aggregates in the presence of 10–15 mg/L of kanamycin (Km) for four months and established several Km-resistant lines as described [19]. The semiquantitative RT-PCR has shown that the *npt*II gene was transcribed in all obtained transgenic cell lines, and the absence of *A. tumefaciens* was confirmed using RT-PCR to control the presence or absence of the *VirB2* gene (Supplementary Figure S1). The selected transformed calli represented friable vigorously growing homogenous tissues, which did not undergo differentiation on the W_B/A_ medium supplemented with 6-benzylaminopurine (BAP) and α-naphthaleneacetic acid (NAA) in the dark. For further analysis, we used 16 transgenic cell lines transformed with the *VaCML* genes: three *VaCML52*-transformed cell lines (52-1, 52-2, 52-3), four *VaCML65*-transformed cell lines (65-1, 65-2, 65-3, 65-4), three *VaCML86*-transformed cell lines (86-1, 86-2, 86-3), three *VaCML93*-transformed cell lines (93-1, 93-2, 93-3), and four *VaCML95*-transformed cell lines (95-1, 95-2, 95-3, 95-4) (Table 1). The control KA0 transformed calli reproduced morphological, growth, and biosynthetic characteristics of the parent V7 culture.

**Figure 1:**
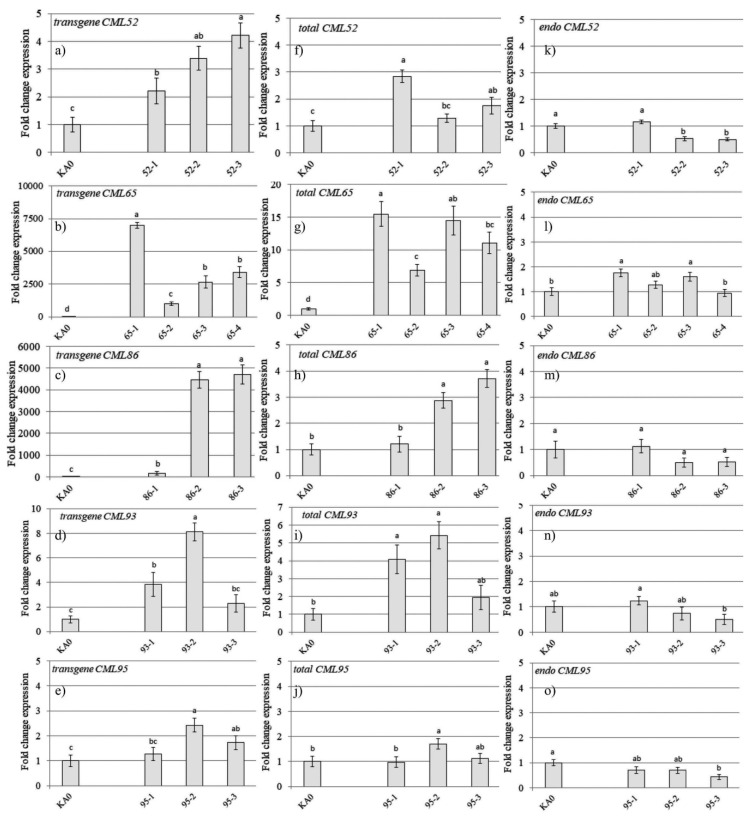
Quantification of the transgene (**a**–**e**), total (**f**–**j**), and endogenous (**k**–**o**) *VaCML* gene expression in the transgenic cells of *Vitis amurensis* performed by quantitative RT-PCR. RNA was extracted from control *V. amurensis* cell line transformed with the empty vector harboring only *npt*II selective marker (KA0) and *V. amurensis* cell lines transformed with the *VaCML52* (**a**,**f**,**k**, *VaCML52*-1, -2, and -3), *VaCML65* (**b**,**g**,**l**, *VaCML65*-1, -2, -3, and -4), *VaCML86* (**c**,**h**,**m**, *VaCML86*-1, -2, and -3), *VaCML93* (**d**,**i**,**n**, *VaCML93*-1, -2, and -3), and *VaCML95* (**e**,**j**,**o**, *VaCML95*-1, -2, -3, and -4) genes. The data are presented as mean ± SE (two independent experiments with eight technical replicates). Means on each figure followed by the same letter were not different using one-way analysis of variance (ANOVA), followed by the Tukey HSD multiple comparison test.

The *VaCML*-transgenic cell lines were confirmed for expression of the transgene and endogenous *VaCML52*, *65*, *86*, *93*, and *95* expressions by qRT-PCR (Figure 1). Most of the *VaCML*-transformed cell lines actively expressed the transgenes (Figure 1a–e). Additionally, the total expression of *VaCML52*, *65*, *86*, *93*, and *95* genes exceeded that in the KA0 control in most of the *VaCML*-transformed calli, except for *VaCML95* (Figure 1k–o). The analysis of the endogenous *VaCML* expression revealed that expression of endogenous *VaCML86* and *VaCML93* was not affected in all cell lines, while expression of *VaCML52*, *65*, and *95* was altered in a part of the cell lines obtained in comparison with that in the control KA0 (Figure 1f–j). On one side, it is known that plant transgenes and/or plant homologous endogenous genes can be silenced as a result of induction of RNA interference (RNAi) and generation of transgene-derived small interfering RNA (siRNAs) [47,48]. In the course of RNAi, double-stranded RNAs (dsRNAs) are processed by a ribonuclease into siRNAs, which are incorporated in the RNA-induced silencing complex that provides cleavage, destabilization, or hindering translation of any homologous mRNAs [49,50]. On the other side, other studies show that in contrast to a conventional transgene, an endogene-resembling transgene was more stably expressed and poorly processed into small RNAs [51,52]. Therefore, there is a need to verify whether either transgene and/or endogene was silenced and to what degree after plant genetic transformation and establishment of plant transgenic cell cultures. The data obtained in the present work show that expression of the endogenous *VaCML52* and *VaCML95* genes was down-regulated after transformation in the 52-2, 52-3, and 95-3 lines, which suggested that the decrease in endogenous expression, in this case, might be caused by the activation of the RNAi process.

### 2.2. Stilbene Content and Biomass Accumulation in the Grapevine VaCML-Transgenic Cell Lines

It is known that the highest content of stilbenes in callus cell cultures of *V. amurensis* was typical for the 35th day of cultivation [53]. Therefore, cell culture samples were collected from the 35-day-old calli for stilbene extraction and biomass analysis. In Table 1, we presented fresh and dry biomass accumulation in the *VaCML*-transformed cell lines of *V. amurensis*. Transformation of the *V. amurensis* cells with the analyzed *VaCML* genes did not substantially affect the fresh and dry growth parameters in most obtained cell lines, except for the fresh weight of the 93-3 and 95-2 cell lines (Table 1).

**Figure 2:**
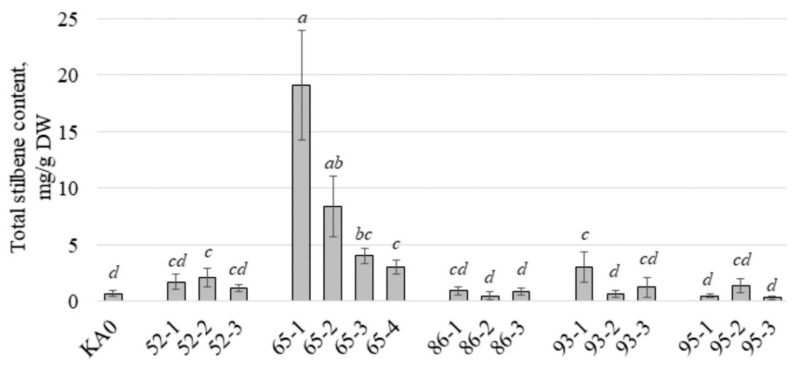
Total stilbene content in the cell lines of *Vitis amurensis* transformed with the *VaCML52*, *VaCML65*, *VaCML86*, *VaCML93*, or *VaCML95* genes in mg per g of the dry weight (DW). KA0—control cell line transformed with the empty vector harboring only *npt*II selective marker; 52-1, 2, 3—cell lines transformed with the *VaCML52* gene; 65-1, 2, 3, 4—cell lines transformed with the *VaCML65* gene; 86-1, 2, 3—cell lines transformed with the *VaCML86* gene; 93-1, 2, 3—cell lines transformed with the *VaCML93* gene; 95-1, 2, 3—cell lines transformed with the *VaCML95* gene. Means followed by the same letter were not different using one-way analysis of variance (ANOVA), followed by the Tukey HSD multiple comparison test (three independent experiments with two technical replicates). *p* < 0.05 was considered statistically significant.

Using HPLC, we determined the content and composition of stilbenes in the obtained *VaCML*-transgenic cell lines. Overexpression of the *VaCML65* gene led to a considerable increase in the content of stilbenes in all obtained transgenic lines in 3.8–23.7 times (Figure 2). Stilbene content in the *VaCML65*-transgenic cell lines reached 19.1 mg per g of the dry cells weight (DW) and stilbene production reached 136 mg per l (Figure 2, Table 1). This is one of the greatest values for cell cultures among the known data ([3], Figure 2, Table 1). This value was approximately 27 times higher than stilbene production in control cell culture KA0 (Table 1) and 4 times higher than stilbene production in *VaCPK20* transgenic cell lines (up to 35 mg/L, [19]), but 1.2 times lower than that in the *rolB* transgenic cell culture of *V. amurensis* (152 mg/L [16]).

Considering other *VaCML* genes, we observed an increase in the content and production of stilbenes in the 52-2 and 93-1 cell lines transformed with the *VaCML52* and *VaCML93* genes (Figure 2; Table 1). We consider this effect rather as a non-specific transformation effect since only one cell line out of three for *VaCML52* or *VaCML93* showed significant changes in the production of stilbenes. All other cell lines transformed with *VaCML52*, *VaCML86*, *VaCML93*, and *VaCML95* showed no significant changes in the content and production of stilbenes (Figure 2; Table 1).

Overexpression of the *VaCML65* gene did not change the spectrum of detected individual stilbenes (Table 2). The increase in the total content of stilbenes in the four *VaCML65*-transgenic cell lines was due to a strong increase in the content of *t*-resveratrol (Table 2). For example, in the *VaCML65*-transgenic lines, the content of *t*-resveratrol increased up to 18.1 mg/g DW, i.e., 4.6–43 times in comparison with resveratrol levels detected in the control KA0 cell line (Table 2). The content of other stilbenoid compounds in the *VaCML65*-transformed cells increased to a lesser extent or even decreased (Table 2). The data indicate that overexpression of the *VaCML65* gene led to the increase in the content of stilbenes via activation of *t*-resveratrol biosynthesis (Table 2).

**Figure 3:**
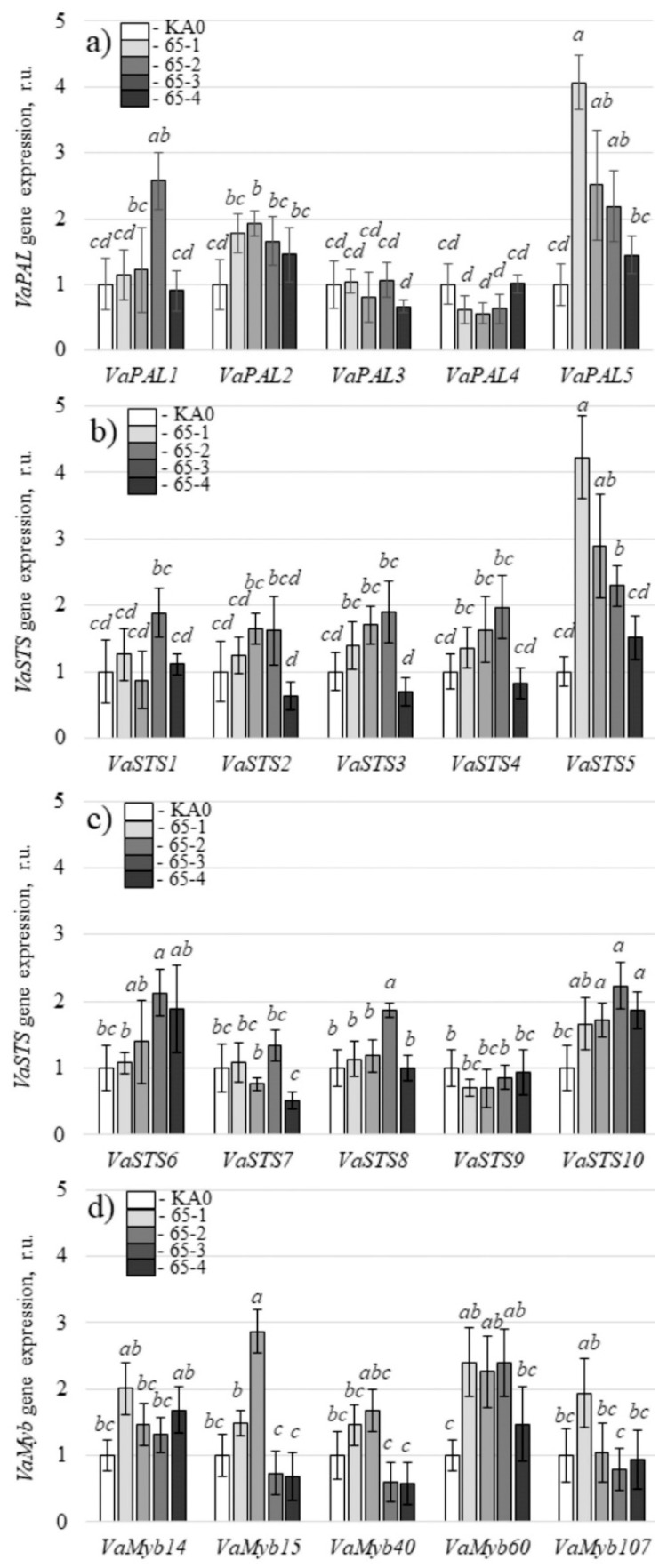
Quantification the *VaPAL1-5* (**a**) and *VaSTS1-5* (**b**), *VaSTS6-10* (**c**), and *VaMyb14*, *15*, *40*, *60*, and *107* (**d**) gene expression in the *VaCML65*-transgenic cell lines of *Vitis amurensis* performed by quantitative PCR (qRT-PCR). RNA was extracted from the vector control (KA0), *VaCML65*-1, -2, -3, and -4-transformed cell lines of *V. amurensis*. Means on each figure followed by the same letter were not different using one-way analysis of variance (ANOVA), followed by the Tukey HSD multiple comparison test (two independent experiments with eight technical replicates). *p* < 0.05 was considered to be statistically significant. n.d.—not detected.

Then, it was important to verify whether the enhanced production of stilbenes in cell cultures overexpressing the *VaCML65* gene was due to the activation of biosynthesis or to a reduction in the degradation of these compounds. For this purpose, we analyzed the expression of several important stilbene biosynthesis genes (Supplementary Figure S2), including five phenylalanine ammonia-lyase (*PAL*), ten stilbene synthase (*STS*), and five genes of *VaMyb14*, *VaMyb15*, *VaMyb40*, *VaMyb60*, and *VaMyb107* transcription factors (Figure 3), which are known as possible positive regulators of stilbene biosynthesis [54]. Expression of these *VaMyb* genes was considerably increased in grapevine cells with a high stilbene content [54].

We showed that overexpression of the *VaCML65* gene led to considerably increased mRNA transcript levels of the *VaPAL1*, *2*, *5* (Figure 3a) and *VaSTS2*, *3*, *4*, *5*, *6*, *8*, and *10* genes (Figure 3b,c) in the four *VaCML65*-transgenic cell lines. The results show that the enhanced content of stilbenes in the obtained *VaCML65*-transgenic grape cells was associated with activation of stilbene biosynthesis via a considerable increase in the expression of certain *PAL* and *STS* genes (Figure 3). Moreover, some Myb transcription factors may be involved in this process, for example, *VaMyb60*, because expression levels of *VaMyb60* increased in all lines after transformation with *VaCML65* gene (Figure 3d).

## 3. Conclusions

In this paper, we investigated the effect of overexpression of five grapevine *CML* genes, which were highly up-regulated in wild-growing grapevine *V. amurensis* in response to stilbene-inducing conditions (stress hormones, phenolic precursors, and UV irradiation), and were suggested as promising candidates playing important roles in stilbene biosynthesis [43]. Previously, it was shown that up-regulation of the *VaCML52*, *VaCML*65, *VaCML93*, and *VaCML95* genes was induced after cultivation in the presence of SA, coumaric acid, MeJA, or UV treatment and associated with an increase in the content of stilbenes in both callus cell cultures and leaves of *V. amurensis* [43]. However, only *VaCML65* overexpression considerably induced stilbene levels in all independent *VaCML65*-transgenic cell lines. The data indicate that the *VaCML65* gene is involved in the signaling pathway regulating stilbene biosynthesis and acts as a positive regulator of *t*-resveratrol production. The proposed model of the signaling pathway leading to stilbene biosynthesis induction with the involvement of VaCML65 in this process was presented in Figure S2. Briefly, after signal perception, stimulated receptors are proposed to induce calcium influx, which then leads to the activation of calcium sensor proteins, including VaCML65. It is possible that VaCML65 together with mitogen-activated protein kinases (MAPK) cascade and hormone signaling could activate expression of *PAL*, *STS,* and other genes responsible for stilbene biosynthesis, probably through activation of some transcription factors (e.g. *VaMyb60*).

The absence of a stable and considerable stilbene-modulating effect in the cell lines of *V. amurensis* overexpressing the *VaCML52*, *VaCML86*, *VaCML93*, and *VaCML95* genes indicates that these *VaCML* genes are not directly involved in the regulation of stilbene biosynthesis in grapevine. It is also possible that additional proteins, which are not present in the cell cultures, are necessary for the work of the *VaCML52*, *VaCML86*, *VaCML93*, and *VaCML95* for their implication as positive regulators for stilbene production. In conclusion, the results are important for understanding the signaling pathways and mechanisms regulating the biosynthesis of plant secondary metabolites and might be in demand in plant biotechnology and agriculture.

## 4. Materials and Methods

### 4.1. Plant Material and Cell Cultures

The V7 callus cultures were established in 2017 from young stems of the wild-growing mature *V. amurensis* vines near Vladivostok as described in Tyunin et al. 2019 [55]. All transgenic cell lines were obtained by *Agrobacterium*-mediated transformation as described in Aleynova-Shumakova et al., 2014 [19]. Briefly, the control KA0 cell culture transformed with the empty vector was obtained in 2020 by co-cultivation of the V7 cell suspension with *Agrobacterium tumefaciens* GV3101:pMP90 strain containing pZP-RCS2-*npt*II [56], which contained only the Km resistance gene, *npt*II.

To generate the construction for plant cell transformation, the full-length cDNA of *VaCML52*, *VaCML65*, *VaCML86*, *VaCML93*, and *VaCML95* genes (GenBank accession number MN540595, MN540606, MN540576, MN540582, MN540584) were amplified by PCR using the primers presented in the Supplementary Table S1. The forward primer contained a *Bgl*II (for *CML52*), *Psp124*B I (or *Sac*I, for *CML65*, *93*, *95*) or *Hind*III (for *CML86*) restriction site and the reverse primer contained a *Bam*HI (for all *CML* genes) restriction site, which are underlined. The full-length cDNA of *CMLs* was cloned into the pSAT1 vector [56] by the *Bgl*II/*Psp124*B I/*Hind*III and *Bam*HI sites under the control of the double cauliflower mosaic virus (CaMV 35S) promoter. Then, the expression cassette from pSAT1 with the *CML* genes was cloned into the pZP-RCS2-*npt*II vector [56] using the *Pal*AI (*Asc*I) sites. The pZP-RCS2-*npt*II construction also carried the *npt*II gene under the control of the double CaMV 35S promoter (Supplementary Figure S3). The used restriction enzymes were obtained from SibEnzyme (Novosibirsk, Russia).

The independently transformed *VaCML*-transgenic callus cell lines of *V. amurensis*, designated 52-1, 52-2, 52-3 (*VaCML52* gene); 65-1, 65-2, 65-3, 65-4 (*VaCML65* gene); 86-1, 86-2, 86-3 (*VaCML86* gene); 93-1, 93-2, 93-3 (*VaCML93* gene); 95-1, 95-2, 95-3, 95-4 (*VaCML95* gene), were obtained in 2020 by transformation of the V7 cell suspension with *A. tumefaciens* strain GV3101::pMP90 containing pZP-RCS2-*VaCML*-*npt*II as described previously [19,57]. The *VaCML* genes and the selective marker *npt*II gene were driven under the double CaMV 35S promoter. Transcript level of the *npt*II gene was verified using semiquantitative RT-PCR with the primers and PCR conditions described earlier [46]. The absence of *A. tumefaciens* was confirmed by RT-PCR of the *VirB2* gene using primers listed in Table S1. All transgenic cell lines were cultivated on the solid Murashige and Skoog modified W_B/A_ medium [58] supplemented with 0.5 mg/L BAP, 2 mg/L NAA, and 8 g/L agar in the dark in 100 mL flasks with 20 mL of the medium. For stilbene analysis, the *V. amurensis* calli were cultivated at 35-day subculture intervals in the dark at 24–25 °C in test tubes (height 150 mm, internal diameter 146 mm) with 7–8 mL of the W_B/A_ medium.

### 4.2. Stilbene Analysis by High-Performance Liquid Chromatography (HPLC) and Mass Spectrometry

The content of stilbenes was measured by HPLC as described [43,59]. Briefly, the extracts were separated on Zorbax C18 column (column temperature 40 °C, 150 mm, 2.1-mm i.d., 3.5-lm part size, Agilent Technologies, Santa Clara, CA, USA) the on HPLC LC-20AD XR analytical system (Shimadzu, Kyoto, Japan). The mobile phase consisted of a gradient elution of 0.1% aqueous acetic acid (A) and acetonitrile (B). The gradient profile with a flow rate of 0.2 mL/min was: 0 min 0% B; 35 min 40% B; 40 min 50% B; 50 min 100% B and then eluent B until 60 min. The injected volume was 5 µL. Liquid chromatography-high-resolution mass spectrometry for quantification of all components was performed using a 1260 Infinity analytical system (Agilent Technologies, USA) as described [20,59]. HPLC for quantification of all components was performed using LC-20 analytical HPLC system (Shimadzu, Japan) equipped with an SPD-M20A photodiode array detector, LC-20ADXR pump, Shim-pack XR-ODS II column and SIL-20ACXR auto sampler as described [46].

### 4.3. RNA Isolation, Reverse Transcription and Quantitative Real-Time PCR (qRT-PCR)

Total RNA extraction was performed using the cetyltrimethylammonium bromide-based extraction as described [47]. Complementary DNAs were synthesized using the MMLV Reverse transcription PCR Kit with oligo(dT)15 (RT-PCR, Evrogen, Moscow, Russia) as described [60]. qRT-PCRs were performed using the real-time PCR kit (Evrogen) and EvaGreen Real-time PCR dye (Biotium, Hayward, CA, USA) using total cDNAs as described [28,46]. The expression was calculated by the 2^−ΔΔCT^ method with two internal controls, *VaGAPDH* and *VaActin1*, as described [61]. The primers used for qRT-PCRs are listed in Table S1. We used different primer sets for analyzing expression of the exogenous (transgene) and endogenous *VaCML* genes. To analyze transcript levels of the *VaCML* transgenes, the primers were designed according to the promoter and terminator sequences of the transgenes (Figure S4; Table S1). To analyze expression of the endogenous *VaCML* genes, the primers were designed to the untranslated (UTR) and specific coding gene regions of the *VaCMLs* (Figure S4; Table S1). To analyze transcript levels of both *VaCML* transgenes and *VaCML* endogenes (total expression), the primers were designed to protein-coding regions of *VaCML* genes (Figure S4; Table S1).

### 4.4. Statistical Analysis

For quantification of the *VaCML*, *VaPAL*, *VaSTS*, and *VaMyb* gene expression we used two independent experiments with eight technical replicates (four qPCR reactions normalized to *VaGAPDH* and four qPCr reactions—to *VaActin* gene in each independent experiment). For callus tissue weight calculations, we used three independent experiments with ten technical replicates and three independent experiments with two technical replicates for total stilbene measurement. The data are presented as mean ± standard error (SE) and were evaluated by one-way analysis of variance (ANOVA), followed by the Tukey HSD multiple comparison test performed in Excel using the XLSTAT software. A value of *p* < 0.05 was considered significant.

## Figures and Tables

**Table 1:** Biomass accumulation and total stilbene production in the cell lines of *Vitis amurensis* overexpressing the *VaCML52*, *VaCML65*, *VaCML86*, *VaCML93*, or *VaCML95* genes.

Cell Line	Overexpressed *CML* Gene	Fresh Weight, g/L	Dry Weight, g/L	Total Stilbene Production, mg/L
KA0	-	163.7 ± 13.3 ^b^	6.65 ± 1.02 ^a^	4.69 ± 1.54 ^d^
52-1	*VaCML52*	155.0 ± 12.8 ^b^	8.07 ± 1.17 ^a^	13.82 ± 5.37 ^c^
52-2		165.1 ± 14.4 ^b^	6.77 ± 0.82 ^a^	14.35 ± 4.88 ^c^
52-3		165.2 ± 10.1 ^b^	7.83 ± 0.97 ^a^	8.93 ± 3.66 ^cd^
65-1	*VaCML65*	131.3 ± 8.5 ^b^	7.11 ± 0.88 ^a^	135.66 ± 34.20 ^a^
65-2		195.1 ± 15.5 ^ab^	6.75 ± 0.91 ^a^	56.67 ± 18.62 ^b^
65-3		137.3 ± 14.9 ^b^	6.91 ± 0.93 ^a^	27.84 ± 13.25 ^b^
65-4		201.1 ± 16.6 ^ab^	8.81 ± 1.32 ^a^	26.94 ± 10.19 ^bc^
86-1	*VaCML86*	158.7 ± 11.2 ^b^	6.69 ± 0.75 ^a^	6.42 ± 1.88 ^d^
86-2		158.9 ± 10.4 ^b^	6.54 ± 0.98 ^a^	3.22 ± 0.91 ^d^
86-3		149.1 ± 8.9 ^b^	5.71 ± 0.74 ^a^	5.05 ± 1.74 ^d^
93-1	*VaCML93*	197.1 ± 13.7 ^ab^	8.75 ± 1.43 ^a^	26.46 ± 11.47 ^bc^
93-2		153.8 ± 12.2 ^b^	6.21 ± 0.88 ^a^	4.31 ± 1.12 ^d^
93-3		218.5 ± 13.4 ^a^	8.77 ± 1.50 ^a^	11.07 ± 5.15 ^c^
95-1	*VaCML95*	198.8 ± 10.8 ^ab^	7.81 ± 0.99 ^a^	3.78 ± 0.74 ^d^
95-2		154.7 ± 11.1 ^b^	6.75 ± 0.79 ^a^	9.28 ± 4.13 ^cd^
95-3		225.2 ± 15.6 ^a^	8.64 ± 1.17 ^a^	2.81 ± 0.65 ^d^

The callus tissue samples were harvested from the 35-day-old cultures (three independent experiments with ten technical replicates for weight calculations and three independent experiments with two technical replicates for total stilbene measurement). Means followed by the same letter in one column were not different using one-way analysis of variance (ANOVA), followed by the Tukey HSD multiple comparison test.

**Table 2:** The content of individual stilbenes (mg per g of the dry weight (DW)) in the transgenic *Vitis amurensis* cell lines transformed with *VaCML52*, *VaCML65*, *VaCML86*, *VaCML93*, or *VaCML95* genes.

Cell Lines	Overexpressed *CML* Gene	*t*-Resveratrol Diglucoside	*t*-Piceid	*t*-Resveratrol	ε-Viniferin	δ-Viniferin	*cis*-Resveratrol	*cis*-Piceid	Piceatannol
KA0	-	0.12 ± 0.07 ^c^	0.09 ± 0.03 ^b^	0.42 ± 0.11 ^f^	0.05 ± 0.02 ^b^	0.12 ± 0.03 ^b^	0 ^a^	0 ^a^	0.01 ± 0.01 ^a^
52-1	*VaCML52*	0.18 ± 0.05 ^c^	0.08 ± 0.03 ^b^	1.06 ± 0.35 ^ef^	0.08 ± 0.04 ^ab^	0.28 ± 0.09 ^ab^	0.01 ± 0.01 ^a^	0.01 ± 0.01 ^a^	0.01 ± 0.01 ^a^
52-2		0.17 ± 0.06 ^c^	0.07 ± 0.03 ^b^	1.62 ± 0.70 ^de^	0.06 ± 0.02 ^b^	0.17 ± 0.05 ^ab^	0 ^a^	0.01 ± 0.01 ^a^	0.01 ± 0.01 ^a^
52-3		0.23 ± 0.09 ^c^	0.07 ± 0.02 ^b^	0.58 ± 0.21 ^f^	0.09 ± 0.03 ^ab^	0.18 ± 0.06 ^ab^	0 ^a^	0 ^a^	0 ^a^
65-1	*VaCML65*	0.26 ± 0.09 ^c^	0.14 ± 0.04 ^b^	18.06 ± 7.09 ^a^	0.12 ± 0.06 ^ab^	0.46 ± 0.16 ^a^	0.01 ± 0.01 ^a^	0.01 ± 0.01 ^a^	0.02 ± 0.01 ^a^
65-2		0.25 ± 0.08 ^c^	0.12 ± 0.03 ^b^	7.79 ± 3.31 ^ab^	0.06 ± 0.03 ^b^	0.17 ± 0.05 ^ab^	0 ^a^	0.01 ± 0.01 ^a^	0.01 ± 0.01 ^a^
65-3		0.72 ± 0.17 ^a^	0.40 ± 0.13 ^a^	2.31 ± 0.47 ^cd^	0.12 ± 0.06 ^ab^	0.25 ± 0.11 ^ab^	0 ^a^	0.01 ± 0.01 ^a^	0.01 ± 0.01 ^a^
65-4		0.68 ± 0.18 ^ab^	0.25 ± 0.08 ^ab^	1.95 ± 0.32 ^cd^	0.08 ± 0.02 ^ab^	0.35 ± 0.14 ^ab^	0 ^a^	0.01 ± 0.01 ^a^	0.01 ± 0.01 ^a^
86-1	*VaCML86*	0.22 ± 0.11 ^c^	0.08 ± 0.03 ^b^	0.33 ± 0.12 ^fg^	0.07 ± 0.02 ^b^	0.25 ± 0.09 ^ab^	0.01 ± 0.01 ^a^	0 ^a^	0.01 ± 0.01 ^a^
86-2		0.13 ± 0.07 ^c^	0.05 ± 0.03 ^b^	0.15 ± 0.04 ^g^	0.08 ± 0.05 ^ab^	0.19 ± 0.11 ^ab^	0 ^a^	0 ^a^	0 ^a^
86-3		0.25 ± 0.11 ^c^	0.08 ± 0.03 ^b^	0.29 ± 0.10 ^fg^	0.05 ± 0.02 ^b^	0.21 ± 0.08 ^ab^	0.01 ± 0.01 ^a^	0.01 ± 0.01 ^a^	0.01 ± 0.01 ^a^
93-1	*VaCML93*	0.14 ± 0.05 ^c^	0.04 ± 0.02 ^b^	2.32 ± 1.03 ^cd^	0.15 ± 0.05 ^a^	0.37 ± 0.11 ^ab^	0.01 ± 0.01 ^a^	0.01 ± 0.01 ^a^	0.01 ± 0.01 ^a^
93-2		0.31 ± 0.12 ^bc^	0.07 ± 0.03 ^b^	0.21 ± 0.09 ^g^	0.02 ± 0.01 ^b^	0.09 ± 0.04 ^b^	0 ^a^	0.02 ± 0.01 ^a^	0^a^
93-3		0.32 ± 0.16 ^bc^	0.11 ± 0.06 ^b^	0.63 ± 0.24 ^f^	0.05 ± 0.03 ^b^	0.17 ± 0.10 ^ab^	0 ^a^	0 ^a^	0 ^a^
95-1	*VaCML95*	0.16 ± 0.06 ^c^	0.06 ± 0.02 ^b^	0.15 ± 0.06 ^g^	0.03 ± 0.01 ^b^	0.10 ± 0.03 ^b^	0.01 ± 0.01 ^a^	0.01 ± 0.01 ^a^	0.01 ± 0.01 ^a^
95-2		0.23 ± 0.07 ^c^	0.05 ± 0.02 ^b^	1.01 ± 0.37 ^ef^	0.03 ± 0.02 ^b^	0.09 ± 0.03 ^b^	0 ^a^	0.01 ± 0.01 ^a^	0.01 ± 0.01 ^a^
95-3		0.11 ± 0.03 ^c^	0.06 ± 0.02 ^b^	0.14 ± 0.07 ^g^	0.02 ± 0.01 ^b^	0.08 ± 0.03 ^b^	0 ^a^	0.01 ± 0.01 ^a^	0 ^a^

KA0—control cell line of *V. amurensis* transformed with the “empty” vector harboring only *npt*II selective marker; 52-1, 2, 3—*V. amurensis* cell lines transformed with the *VaCML52* gene; 65-1, 2, 3, 4—*V. amurensis* cell lines transformed with the *VaCML65* gene; 86-1, 2, 3—*V. amurensis* cell lines transformed with the *VaCML86* gene; 93-1, 2, 3—*V. amurensis* cell lines transformed with the *VaCML93* gene; 95-1, 2, 3—*V. amurensis* cell lines transformed with the *VaCML95* gene. The callus tissue samples were harvested from the 35-day-old cell cultures. Means followed by the same letter in one row were not different using one-way analysis of variance (ANOVA), followed by the Tukey HSD multiple comparison test (three independent experiments with two technical replicates). *p* < 0.05 was considered statistically significant.

## Data Availability

The data presented in this study are available in all Tables and Figures of the manuscript.

## Supplementary Materials

The following supporting information can be downloaded at: https://www.mdpi.com/article/10.3390/plants11020171/s1, Table S1: Primers used for amplification of *Vitis amurensis* cDNAs PCRs. Figure S1: Transcript levels of the *npt*II and *VirB2* genes in grape cell cultures shown using separation of semiquantitative RT-PCR products of the *npt*II and *VirB2* genes by gel electrophoresis. KA0—control cell line of *Vitis amurensis* transformed with the “empty” vector harboring only *npt*II selective marker; 52-1, 2, 3—*V. amurensis* cell lines transformed with the *VaCML52* gene; 65-1, 2, 3, 4—*V. amurensis* cell lines transformed with the *VaCML65* gene; 86-1, 2, 3—*V. amurensis* cell lines transformed with the *VaCML86* gene; 93-1, 2, 3—*V. amurensis* cell lines transformed with the *VaCML93* gene; 95-1, 2, 3—*V. amurensis* cell lines transformed with the *VaCML95* gene. Pc—positive control (pZP-RCS-*npt*II for *npt*II or *Agrobacterium tumefaciens* strain GV3101:pMP90 for *VirB2*); Nc—negative control (PCR mixture without DNA or bacteria). Figure S2: Proposed model of the signaling pathway leading to stilbene biosynthesis induction and VaCML65 functions in this process in the grapevine cells. Ultraviolet (UV), elicitors, pathogen effectors, and other environmental cues are perceived by specific receptors. Recognition of an external signal leads to sustained calcium influx, activation of VaCML65 and other calcium sensors, MAPK cascades, which promotes plant hormone signaling and TF activation. TFs activate transcription of the genes responsible for stilbene biosynthesis. MAPK—mitogen activated protein kinases; CMLs—calmodulin-like proteins; TF—transcription factors. Figure S3: Schematic representation of the pZP-RCS2-*npt*II-*VaCML* vector [46] carrying the *VaCML* genes. RB and LB—correspond to the sequences of the right and left borders of T-DNA; 2*35S—the double 35S promoter of the cauliflower mosaic virus (CaMV); ter—CaMV 35S terminator; *npt*II—kanamycin resistance gene; Sp—spectinomycin resistance gene. Figure S4: Schematic representation of the T-DNA region from the pZP-RCS2-*npt*II-*VaCML* vector (a) and the endogenous *VaCML* gene of *Vitis amurensis* (b) with the used primer sets for analyzing the transgene *VaCML* and endogenous *VaCML* transcript levels. 2*35S—the double 35S promoter of the cauliflower mosaic virus (CaMV); ter—CaMV 35S terminator; *VaCML*—the coding sequence of the *VaCML52*, *VaCML65*, *VaCML86*, *VaCML93*, or *VaCML95* genes; 5’UTR and 3’UTR—untranslated regions of the *VaCML* genes. CML-S and pSAT-term-A—primers for qRT-PCR estimation of the transgene *VaCML52*, *65*, *86*, and *93* expression; pSAT-prom-S and CML95-A—primers for qRT-PCR estimation of the transgene *VaCML95* expression; CML-S and CML-endo-A—primers for qRT-PCR estimation of the endogenous *VaCML52*, *65*, *86*, and *93* expression; CML-endo-S and CML95-A—primers for qRT-PCR estimation of the endogenous *VaCML95* expression; CML-S and CML-A—primers for qRT-PCR estimation of the total *VaCML52*, *65*, *86*, and *93* expression; CML95-S and CML95-A—primers for qRT-PCR estimation of the total *VaCML95* expression.

## References

[1] Langcake P., Pryce R.J. (1977). A new class of phytoalexins from grapevines. Experientia.

[2] Kiselev K.V. (2011). Perspectives for production and application of resveratrol. Appl. Microbiol. Biotechnol..

[3] Dubrovina A.S., Kiselev K.V. (2017). Regulation of stilbene biosynthesis in plants. Planta.

[4] Colica C., Milanovic M., Milic N., Aiello V., De Lorenzo A., Abenavoli L. (2018). A systematic review on natural antioxidant properties of resveratrol. Nat. Prod. Commun..

[5] Shaito A., Posadino A.M., Younes N., Hasan H., Halabi S., Alhababi D., Al-Mohannadi A., Abdel-Rahman W.M., Eid A.H., Nasrallah G.K. (2020). Potential adverse effects of resveratrol: A literature review. Int. J. Mol. Sci..

[6] Sikuten I., Stambuk P., Andabaka Z., Tomaz I., Markovic Z., Stupic D., Maletic E., Kontic J.K., Preiner D. (2020). Grapevine as a rich source of polyphenolic compounds. Molecules.

[7] Jeandet P., Delaunois B., Conreux A., Donnez D., Nuzzo V., Cordelier S., Clément C., Courot E. (2010). Biosynthesis, metabolism, molecular engineering, and biological functions of stilbene phytoalexins in plants. Biofactors.

[8] Riccio B.V.F., Fonseca-Santos B., Ferrari P.C., Chorilli M. (2020). Characteristics, biological properties and analytical methods of *trans*-resveratrol: A review. Crit. Rev. Anal. Chem..

[9] Chong J.L., Poutaraud A., Hugueney P. (2009). Metabolism and roles of stilbenes in plants. Plant Sci..

[10] Faurie B., Cluzet S., Mérillon J.M. (2009). Implication of signaling pathways involving calcium, phosphorylation and active oxygen species in methyl jasmonate-induced defense responses in grapevine cell cultures. J. Plant Physiol..

[11] Vandelle E., Vannozzi A., Wong D., Danzi D., Digby A.M., Dal Santo S., Astegno A. (2018). Identification, characterization, and expression analysis of calmodulin and calmodulin-like genes in grapevine (*Vitis vinifera*) reveal likely roles in stress responses. Plant Physiol. Biochem..

[12] Belchi-Navarro S., Almagro L., Sabater-Jara A.B., Fernandez-Perez F., Bru R., Pedreno M.A. (2013). Induction of *trans*-resveratrol and extracellular pathogenesis-related proteins in elicited suspension cultured cells of *Vitis vinifera* cv Monastrell. J. Plant Physiol..

[13] Nicolas P., Lecourieux D., Kappel C., Cluzet S., Cramer G., Delrot S., Lecourieux F. (2014). The basic leucine zipper transcription factor ABSCISIC ACID RESPONSE ELEMENT-BINDING FACTOR2 is an important transcriptional regulator of abscisic acid-dependent grape berry ripening processes. Plant Physiol..

[14] Xu A., Zhan J.C., Huang W.D. (2015). Effects of ultraviolet C, methyl jasmonate and salicylic acid, alone or in combination, on stilbene biosynthesis in cell suspension cultures of *Vitis vinifera* L. cv. Cabernet Sauvignon. Plant Cell Tissue Organ Cult..

[15] Degu A., Ayenew B., Cramer G.R., Fait A. (2016). Polyphenolic responses of grapevine berries to light, temperature, oxidative stress, abscisic acid and jasmonic acid show specific developmental-dependent degrees of metabolic resilience to perturbation. Food Chem..

[16] Dubrovina A.S., Kiselev K.V., Veselova M.V., Isaeva G.A., Fedoreyev S.A., Zhuravlev Y.N. (2009). Enhanced resveratrol accumulation in *rolB* transgenic cultures of *Vitis amurensis* correlates with unusual changes in *CDPK* gene expression. J. Plant Physiol..

[17] Kiselev K.V., Shumakova O.A., Manyakhin A.Y., Mazeika A.N. (2012). Influence of calcium influx induced by the calcium ionophore, A23187, on resveratrol content and the expression of *CDPK* and *STS* genes in the cell cultures of *Vitis amurensis*. Plant Growth Regul..

[18] Kiselev K.V., Shumakova O.A., Manyakhin A.Y. (2013). Effects of the calmodulin antagonist W7 on resveratrol biosynthesis in *Vitis amurensis* Rupr.. Plant Mol. Biol. Rep..

[19] Aleynova-Shumakova O.A., Dubrovina A.S., Manyakhin A.Y., Karetin Y.A., Kiselev K.V. (2014). *VaCPK20* gene overexpression significantly increased resveratrol content and expression of stilbene synthase genes in cell cultures of *Vitis amurensis* Rupr.. Appl. Microbiol. Biotechnol..

[20] Aleynova O.A., Dubrovina A.S., Manyakhin A.Y., Karetin Y.A., Kiselev K.V. (2015). Regulation of resveratrol production in *Vitis amurensis* cell cultures by calcium-dependent protein kinases. Appl. Biochem. Biotechnol..

[21] Hoell J., Vannozzi A., Czemmel S., D’Onofrio C., Walker A.R., Rausch T., Lucchin M., Boss P.K., Dry I.B., Bogs J. (2013). The R2R3-MYB transcription factors MYB14 and MYB15 regulate stilbene biosynthesis in *Vitis vinifera*. Plant Cell.

[22] Fang L., Hou Y., Wang L., Xin H., Wang N., Li S. (2014). Myb14, a direct activator of *STS*, is associated with resveratrol content variation in berry skin in two grape cultivars. Plant Cell Rep..

[23] Kiselev K.V., Aleynova O.A., Ogneva Z.V., Suprun A.R., Ananev A.A., Nityagovsky N.N., Dubrovina A.S. (2021). The effect of stress hormones, UV-C, and stilbene precursors on calmodulin (*CaM*) and calmodulin-like gene (*CML*) expression in *Vitis amurensis* Rupr.. Plant Cell Tissue Organ Cult..

[24] Choi W.G., Hilleary R., Swanson S.J., Kim S.H., Gilroy S. (2016). Rapid, long-distance electrical and calcium signaling in plants. Annu. Rev. Plant Biol..

[25] Kudla J., Becker D., Grill E., Hedrich R., Hippler M., Kummer U., Parniske M., Romeis T., Schumacher K. (2018). Advances and current challenges in calcium signaling. New Phytol..

[26] DeFalco T.A., Bender K.W., Snedden W.A. (2010). Breaking the code: Ca^2+^ sensors in plant signaling. Biochem. J..

[27] Mohanta T.K., Yadav D., Khan A.L., Hashem A., Abd Allah E.F., Al-Harrasi A. (2019). Molecular players of EF-hand containing calcium signaling event in plants. Int. J. Mol. Sci..

[28] McCormack E., Braam J. (2003). Calmodulins and related potential calcium sensors of Arabidopsis. New Phytol..

[29] Dubrovina A.S., Aleynova O.A., Ogneva Z.V., Suprun A.R., Ananev A.A., Kiselev K.V. (2019). The effect of abiotic stress conditions on expression of calmodulin (*CaM*) and calmodulin-like (*CML*) genes in wild-growing grapevine *Vitis amurensis*. Plants.

[30] Batistič O., Kudla J. (2012). Analysis of calcium signaling pathways in plants. Biochim. Biophys. Acta.

[31] Dobney S., Chiasson D., Lam P., Smith S.P., Snedden W.A. (2009). The calmodulin-related calcium sensor CML42 plays a role in trichome branching. J. Biol. Chem..

[32] Cheval C., Aldon D., Galaud J.P., Ranty B. (2013). Calcium/calmodulin-mediated regulation of plant immunity. Biochim. Biophys. Acta.

[33] Zeng H., Xu L., Singh A., Wang H., Du L., Poovaiah B.W. (2015). Involvement of calmodulin and calmodulin-like proteins in plant responses to abiotic stresses. Front. Plant Sci..

[34] Midhat U., Ting M.K.Y., Teresinski H.J., Snedden W.A. (2018). The calmodulin-like protein, CML39, is involved in regulating seed development, germination, and fruit development in *Arabidopsis*. Plant Mol. Biol..

[35] Shen L., Yang S., Guan D.Y., He S.L. (2020). CaCML13 acts positively in pepper immunity against *Ralstonia solanacearum* infection forming feedback loop with CabZIP63. Int. J. Mol. Sci..

[36] Vanderbeld B., Snedden W.A. (2007). Developmental and stimulus-induced expression patterns of Arabidopsis calmodulin-like genes *CML37*, *CML38* and *CML39*. Plant Mol. Biol..

[37] Bender K.W., Dobney S., Ogunrinde A., Chiasson D., Mullen R.T., Teresinski H.J., Singh P., Munro K., Smith S.P., Snedden W.A. (2014). The calmodulin-like protein CML43 functions as a salicylic-acid-inducible root-specific Ca^2+^ sensor in Arabidopsis. Biochem. J..

[38] Lu L., Rong W., Zhou R., Huo N., Zhang Z. (2019). TaCML36, a wheat calmodulin-like protein, positively participates in an immune response to *Rhizoctonia cerealis*. Crop J..

[39] Wan D.L., Li R.L., Zou B., Zhang X., Cong J.Y., Wang R.G., Xia Y.J., Li G.J. (2012). Calmodulin-binding protein CBP60g is a positive regulator of both disease resistance and drought tolerance in Arabidopsis. Plant Cell Rep..

[40] Rao S.S., El-Habbak M.H., Havens W.M., Singh A., Zheng D.M., Vaughn L., Haudenshield J.S., Hartman G.L., Korban S.S., Ghabrial S.A. (2014). Overexpression of *GmCaM4* in soybean enhances resistance to pathogens and tolerance to salt stress. Mol. Plant Pathol..

[41] Munir S., Liu H., Xing Y., Hussain S., Ouyang B., Zhang Y., Li H., Ye Z. (2016). Overexpression of calmodulin-like (*ShCML44*) stress-responsive gene from *Solanum habrochaites* enhances tolerance to multiple abiotic stresses. Sci. Rep..

[42] Kalaipandian S., Xue G.P., Rae A.L., Glassop D., Bonnett G.D., McIntyre L.C. (2019). Overexpression of *TaCML20*, a calmodulin-like gene, enhances water soluble carbohydrate accumulation and yield in wheat. Physiol. Plant..

[43] Ma Q.P., Zhou Q.Q., Chen C.M., Cui Q.Y., Zhao Y.X., Wang K., Arkorful E., Chen X., Sun K., Li X.H. (2019). Isolation and expression analysis of *CsCML* genes in response to abiotic stresses in the tea plant (*Camellia sinensis*). Sci. Rep..

[44] Ma Y.Y., Zhang Y.L., Shao H., Lu J. (2010). Differential physio-biochemical responses to cold stress of cold-tolerant and non-tolerant grapes (*Vitis* L.) from China. J. Agron. Crop Sci..

[45] Liu L., Li H. (2013). Review research progress in amur grape, *Vitis amurensis* Rupr.. Can. J. Plant Sci..

[46] Kiselev K.V., Aleynova O.A., Grigorchuk V.P., Dubrovina A.S. (2017). Stilbene accumulation and expression of stilbene biosynthesis pathway genes in wild grapevine *Vitis amurensis* Rupr.. Planta.

[47] Eamens A., Wang M.B., Smith N.A., Waterhouse P.M. (2008). RNA silencing in plants: Yesterday, today, and tomorrow. Plant Physiol..

[48] Velten J., Cakir C., Youn E., Chen J., Cazzonelli C.I. (2012). Transgene silencing and transgene-derived siRNA production in tobacco plants homozygous for an introduced *AtMYB90* construct. PLoS ONE.

[49] Wilson R.C., Doudna J.A. (2013). Molecular mechanisms of RNA interference. Annu. Rev. Biophys..

[50] Rosa C., Kuo Y.W., Wuriyanghan H., Falk B.W. (2018). RNA interference mechanisms and applications in plant pathology. Annu. Rev. Phytopathol..

[51] Dadami E., Moser M., Zwiebel M., Krczal G., Wassenegger M., Dalakouras A. (2013). An endogene-resembling transgene delays the onset of silencing and limits siRNA accumulation. FEBS Lett..

[52] Dadami E., Dalakouras A., Zwiebel M., Krczal G., Wassenegger M. (2014). An endogene-resembling transgene is resistant to DNA methylation and systemic silencing. RNA Biol..

[53] Suprun A.R., Ogneva Z.V., Dubrovina A.S., Kiselev K.V. (2020). Effect of spruce *PjSTS1a*, *PjSTS2*, or *PjSTS3* gene overexpression on stilbene biosynthesis in callus cultures of *Vitis amurensis* Rupr.. Biotechnol. Appl. Biochem..

[54] Kiselev K.V., Aleynova O.A., Tyunin A.P. (2017). Expression of the *R2R3 MYB* transcription factors in *Vitis amurensis* Rupr. plants and cell cultures with different resveratrol content. Russ. J. Genet..

[55] Tyunin A.P., Suprun A.R., Nityagovsky N.N., Manyakhin A.Y., Karetin Y.A., Dubrovina A.S., Kiselev K.V. (2019). The effect of explant origin and collection season on stilbene biosynthesis in cell cultures of *Vitis amurensis* Rupr.. Plant Cell Tissue Organ Cult..

[56] Tzfira T., Tian G.W., Lacroix B., Vyas S., Li J., Leitner-Dagan Y., Krichevsky A., Taylor T., Vainstein A., Citovsky V. (2005). pSAT vectors: A modular series of plasmids for autofluorescent protein tagging and expression of multiple genes in plants. Plant Mol. Biol..

[57] Kiselev K.V., Dubrovina A.S., Shumakova O.A., Karetin Y.A., Manyakhin A.Y. (2013). Structure and expression profiling of a novel calcium-dependent protein kinase gene, *CDPK3a*, in leaves, stems, grapes, and cell cultures of wild-growing grapevine *Vitis amurensis* Rupr.. Plant Cell Rep..

[58] Dubrovina A.S., Manyakhin A.Y., Zhuravlev Y.N., Kiselev K.V. (2010). Resveratrol content and expression of phenylalanine ammonia-lyase and stilbene synthase genes in *rolC* transgenic cell cultures of *Vitis amurensis*. Appl. Microbiol. Biotechnol..

[59] Aleynova O.A., Grigorchuk V.P., Dubrovina A.S., Rybin V.G., Kiselev K.V. (2016). Stilbene accumulation in cell cultures of *Vitis amurensis* Rupr. overexpressing *VaSTS1*, *VaSTS2*, and *VaSTS7* genes. Plant Cell Tissue Organ Cult..

[60] Kiselev K.V., Ogneva Z.V., Suprun A.R., Grigorchuk V.P., Dubrovina A.S. (2019). Action of ultraviolet-C radiation and p-coumaric acid on stilbene accumulation and expression of stilbene biosynthesis-related genes in the grapevine *Vitis amurensis* Rupr.. Acta Physiol. Plant.

[61] Livak K.J., Schmittgen T.D. (2001). Analysis of relative gene expression data using real-time quantitative PCR and the 2^−ΔΔCT^ method. Methods.

