# Supplementary material for: Effect of Calmodulin-like Gene (CML) Overexpression on Stilbene Biosynthesis in Cell Cultures of Vitis amurensis Rupr
Source: Plants (Basel). 2022 Jan 10;11(2):171. doi: 10.3390/plants11020171 (PMC8778512; doi:10.3390/plants11020171)

Supplementary Table S1.

Primers used for amplification of *Vitis amurensis* cDNAs in PCR.

cDNA	Primers names	Primers sequences, 5'-3'
Primers for real-time PCRs		
VaCML52 transgene<br>(MN540595)	*VaCML52*-S<br>pSAT-term-A	GGAGACCATTTCATTAGCGA<br>GAGAGACTGGTGATTTTTGCG
VaCML52 endogene<br>(MN540595)	*VaCML52*-S<br>*VaCML52*-endo-A	GGAGACCATTTCATTAGCGA<br>AAAAGCCTAAAACCTTTCTTGTAAGA
VaCML52 total<br>(MN540595)	*VaCML52*-S<br>*VaCML52*-A	GGAGACCATTTCATTAGCGA<br>CTAACGTAACTTAAATCCCCAT
VaCML65 transgene<br>(MN540606)	*VaCML65*-S<br>pSAT-term-A	GCTTCCTGAAGAAAGCTGTT<br>GAGAGACTGGTGATTTTTGCG
VaCML65 endogene<br>(MN540606)	*VaCML65*-S<br>*VaCML65*-endo-A	GCTTCCTGAAGAAAGCTGTT<br>ATACCTCAACAAAGTCAACAAACA
VaCML65 total<br>(MN540606)	*VaCML65*-S<br>*VaCML65*-A	GCTTCCTGAAGAAAGCTGTT<br>TCAAGCATCCATTTTCATGG
VaCML86 transgene<br>(MN540576)	*VaCML86*-S<br>pSAT-term-A	GGATCTTTTGACTCCGACG<br>GAGAGACTGGTGATTTTTGCG
VaCML86 endogene<br>(MN540576)	*VaCML86*-S<br>*VaCML86*-endo-A	GGATCTTTTGACTCCGACG<br>AAGAATCCATGCGCCTCAGCAG
VaCML86 total<br>(MN540576)	*VaCML86*-S<br>*VaCML86*-A	GGATCTTTTGACTCCGACG<br>TCACTGTTGACGCTGTTTAG
VaCML93 transgene<br>(MN540582)	*VaCML93*-S<br>pSAT-term-A	GTTGCATCACCCCAAAGAGCTTG<br>GAGAGACTGGTGATTTTTGCG
VaCML93 endogen<br>(MN540582)	*VaCML93*-S<br>*VaCML93*-endo-A	GTTGCATCACCCCAAAGAGCTTG<br>GGAAAGCAAGGCATGATGATC
VaCML93 total<br>(MN540582)	*VaCML93*-S<br>*VaCML93*-A	GTTGCATCACCCCAAAGAGCTTG<br>ATCGATGCTCCGGGATTCACCA
VaCML95 transgene<br>(MN540584)	pSAT-prov-S<br>*VaCML95*-A	CAAGCATTCTACTTCTATTG<br>CGCCTCCTCCATCAGCAG
VaCML95 endogen<br>(MN540584)	*VaCML95*-endo-S<br>*VaCML95*-A	CAAGTTCTTGCGGTAAGCAGC<br>CGCCTCCTCCATCAGCAG
VaCML95 total<br>(MN540584)	*VaCML95*-S<br>*VaCML95*-A	GGTGATGGGAAACTCTCTC<br>CGCCTCCTCCATCAGCAG
VaActin1<br>(DQ517935)	*VaActin*-realS<br>*VaActin*-realA	GTATTGTGCTGGATTCTGGTGA<br>GCAAGGTCAAGACGAAGGATAG
VaGAPDH<br>(AM437491)	*VaGAPDH*-realS<br>*VaGAPDH*-realA	CACTGAAGATGATGTTGTTTCC<br>GCTATTCCAGCCTTGGCAT
Primers for generation the construction for plant cell transformation		
Overexpression of<br>*VaCML52*	*VaCml52*-Bgl<br>*VaCml52*-BamH	ACTCAGATCTATGCCTCTGTGGACTCCGA<br>TCGAGGATCCTCACTAACGTAACTTAAA
Overexpression of<br>*VaCML65*	*VaCml65*-Psp<br>*VaCml65*-BamH	ACTCGAGCTCATGAGTGTGGAAGTGTTGG<br>TCGAGGATCCTCATCAAGCATCCATTTTC
Overexpression of<br>*VaCML86*	*VaCml86*-Hind<br>*VaCml86*-BamH	GCTCAAGCTTATGACGAGCAATTCTATTTC<br>TCGAGGATCCTTACTGTTGACGCTGTTTAGA
Overexpression of<br>*VaCML93*	*VaCml93*-Psp<br>*VaCml93*-BamH	ACTCGAGCTCATGGCAAACGATGAGATG<br>TCGAGGATCCTCACTATAACATCATGACC
Overexpression of<br>*VaCML95*	*VaCml95*-Psp<br>*VaCml95*-BamH	ACTCGAGCTCATGATTAACTGCAGCATAT<br>TCGAGGATCCTCATCAAAACATCATGAGC
Primers for semiquantitative RT-PCR		
*npt*II (AY818371)	NPTII-SER-S<br>NPTII-KON-A	ATTCGACCACCAAGCGAAAC<br>TCAGAAGAACTCGTCAAGAA
VirB2 (KY000054)	VIRB2-S<br>VIRB2-A	ATGCGATGCTTTGAAAGATACCG<br>TTAGCCACCTCCAGTCAGCG

Supplementary Fig. S1.

Transcript levels of the *npt*II and *VirB2* genes in grape cell cultures shown using separation of semiquantitative RT-PCR products of the *npt*II and *VirB2* genes by gel electrophoresis. KA0 – control cell line of *Vitis amurensis* transformed with the "empty" vector harboring only *npt*II selective marker; 52-1, 2, 3 – *V. amurensis* cell lines transformed with the *VaCML52* gene; 65-1, 2, 3, 4 – *V. amurensis* cell lines transformed with the *VaCML65* gene; 86-1, 2, 3 – *V. amurensis* cell lines transformed with the *VaCML86* gene; 93-1, 2, 3 – *V. amurensis* cell lines transformed with the *VaCML93* gene; 95-1, 2, 3 – *V. amurensis* cell lines transformed with the *VaCML95* gene. Pc - positive control (pZP-RCS-*npt*II for *npt*II or *Agrobacterium tumefaciens* strain GV3101:pMP90 for *VirB2*); Nc - negative control (PCR mixture without DNA or bacteria).

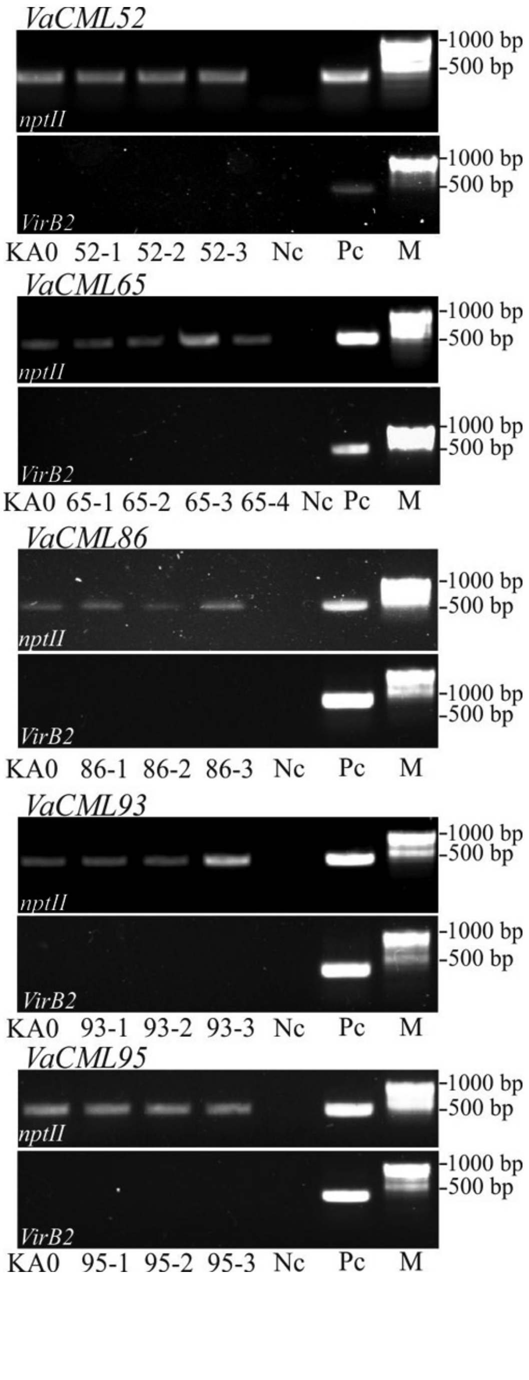

Supplementary Fig. S2.

Proposed model of the signaling pathway leading to stilbene biosynthesis induction and VaCML65 functions in this process in the grapevine cells. Ultraviolet (UV), elicitors, pathogen effectors, and other environmental cues are perceived by specific receptors. Recognition of an external signal leads to sustained calcium influx, activation of VaCML65 and other calcium sensors, MAPK cascades, which promotes plant hormone signaling and TF activation. TFs activate transcription of the genes responsible for stilbene biosynthesis. MAPK – mitogen activated protein kinases; CMLs – calmodulin-like proteins; TF - transcription factors.

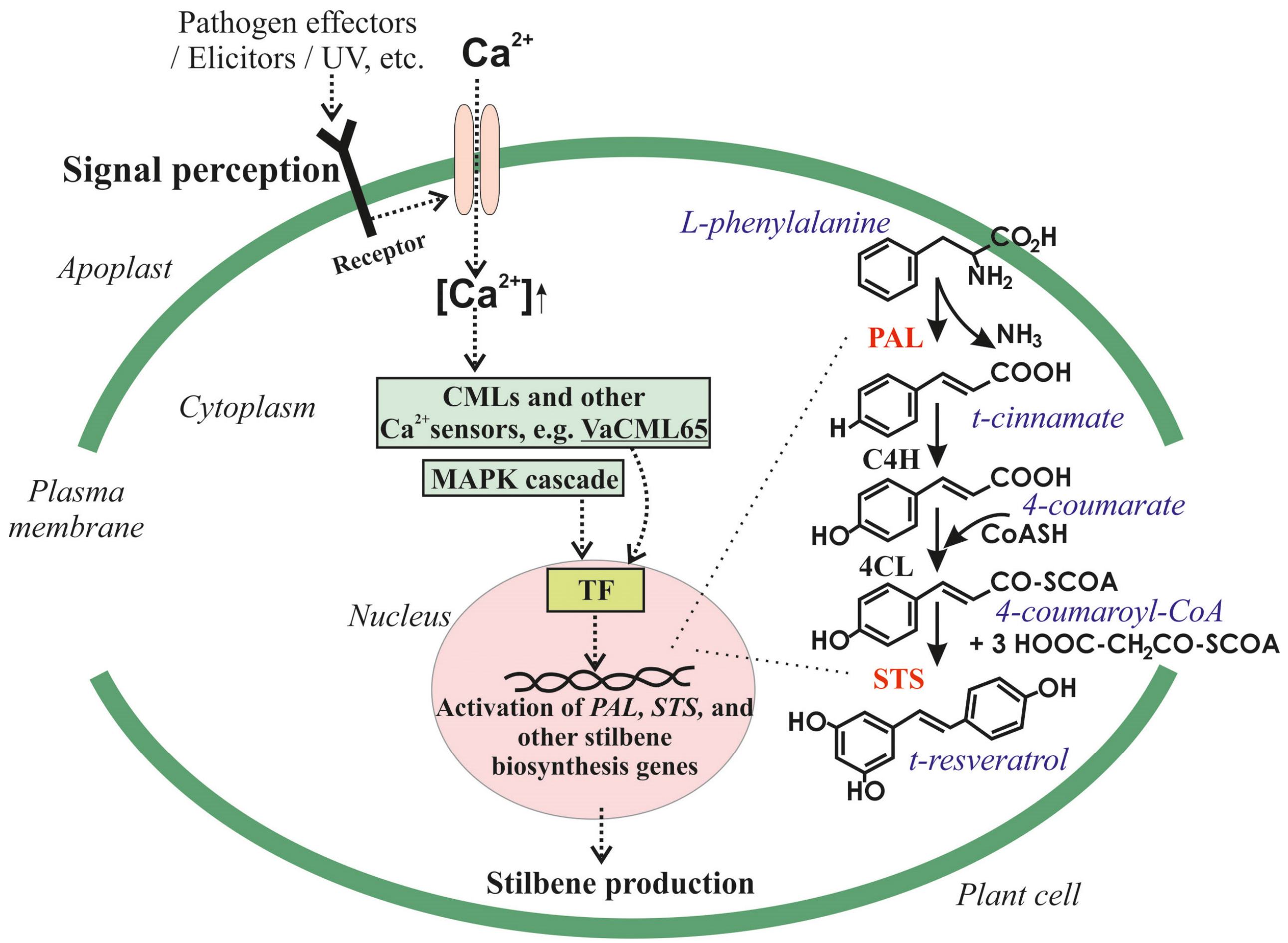

Supplementary Fig. S3.
Schematic representation of the pZP-RCS2-*nptII-VaCML* vector [46] carrying the *VaCML* genes. RB and LB – correspond to the sequences of the right and left borders of T-DNA; 2*35S – the double 35S promoter of the cauliflower mosaic virus (CaMV); ter - CaMV 35S terminator; *nptII* - kanamycin resistance gene; Sp - spectinomycin resistance gene.

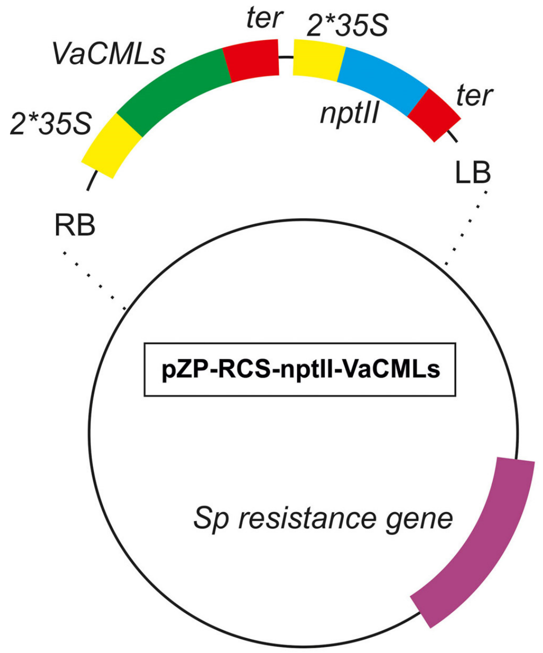

Supplementary Fig. S4.
Schematic representation of the T-DNA region from the pZP-RCS2-*nptII-VaCML* vector (a) and the endogenous *VaCML* gene of *Vitis amurensis* (b) with the used primer sets for analyzing the transgene *VaCML* and endogenous *VaCML* transcript levels. 2*35S – the double 35S promoter of the cauliflower mosaic virus (CaMV); ter - CaMV 35S terminator; *VaCML* – the coding sequence of the *VaCML52*, *VaCML65*, *VaCML86*, *VaCML93*, or *VaCML95* genes; 5'UTR and 3'UTR – untranslated regions of the *VaCML* genes. CML-S and pSAT-term-A – primers for qRT-PCR estimation of the transgene *VaCML52*, *65*, *86*, and *93* expression; pSAT-prom-S and CML95-A – primers for qRT-PCR estimation of the transgene *VaCML95* expression; CML-S and CML-endo-A – primers for qRT-PCR estimation of the endogenous *VaCML52*, *65*, *86*, and *93* expression; CML-endo-S and CML95-A – primers for qRT-PCR estimation of the endogenous *VaCML95* expression; CML-S and CML-A – primers for qRT-PCR estimation of the total *VaCML52*, *65*, *86*, and *93* expression; CML95-S and CML95-A – primers for qRT-PCR estimation of the total *VaCML95* expression.

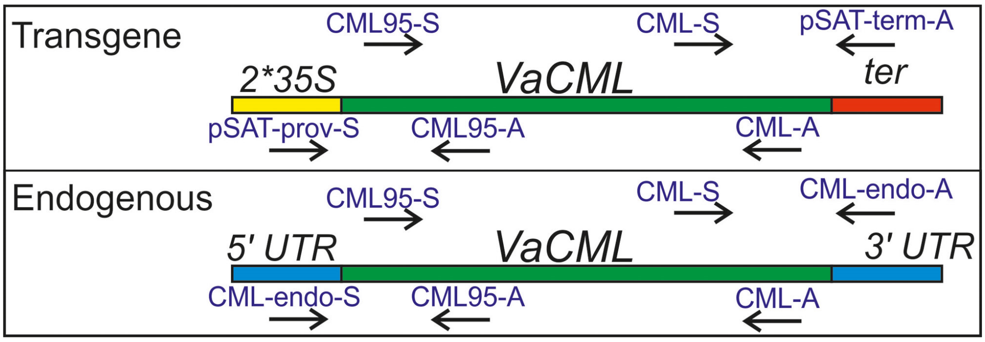

Supplement: Supplementary file 1 [file plants-11-00171-s001.zip › plants-1552414-supplementary.pdf]
